# Access to oncology care in Mali: a qualitative study on breast cancer

**DOI:** 10.1186/s12885-024-11825-6

**Published:** 2024-01-15

**Authors:** Clémence Schantz, Abdourahmane Coulibaly, Alassane Traoré, Bakary Abou Traoré, Kadiatou Faye, Julie Robin, Luis Teixeira, Valéry Ridde, and the SENOVIE group, Moufalilou Aboubakar, Myriam Baron, Gaëtan Des Guetz, Anne Gosselin, Hamidou Niangaly, Emmanuel Bonnet, Fanny Chabrol, Justin Lewis Denakpo, Annabel Desgrées du Loû, Freddy Gnangnon, Pascale Hancart Petitet, Joseph Larmarange, Dolorès Pourette, Léa Prost, Beauta Rath, Priscille Sauvegrain, Angéline Tonato Bagnan

**Affiliations:** 1 Université Paris Cité, IRD, Inserm, Ceped, F-75 006 Paris, France; 2 Institution Convergences Et Migrations, Aubervilliers, France; 3 Faculté de Médecine Et d’Odontostomatologie de Bamako, Bamako, Mali; 4 Hôpital du Mali, Bamako, Mali; 5 Centre de Santé de Référence de La Commune 2, Bamako, Mali; 6 Centre National de La Recherche Scientifique Et Technologie (CNRST), Bamako, Mali; 7 Association Les Combattantes du Cancer, Bamako, Mali; 8 Pathophysiology of Breast Cancer Team, Department of Senology, Université Paris Cité INSERM U976, HIPIAP-HP, Hôpital Saint-Louis, Paris, France

**Keywords:** Cancer, Mali, Access to care, Barriers, Opportunities

## Abstract

**Background:**

Breast cancer is the most common cancer in terms of incidence and mortality among women worldwide, including in Africa, and a rapid increase in the number of new cases of breast cancer has recently been observed in sub-Saharan Africa. Oncology is a relatively new discipline in many West African countries, particularly Mali; thus, little is known about the current state of cancer care infrastructure and oncology practices in these countries.

**Methods:**

To describe the challenges related to access to oncology care in Mali, we used a qualitative approach, following the Consolidated Criteria for Reporting Qualitative Research (COREQ). Thirty-eight semistructured interviews were conducted with health professionals treating cancer in Mali (*n* = 10), women with breast cancer (*n* = 25), and representatives of associations (*n* = 3), and 40 participant observations were conducted in an oncology unit in Bamako. We used the theoretical framework on access to health care developed by Levesque et al. a posteriori to organise and analyse the data collected.

**Results:**

Access to oncology care is partly limited by the current state of Mali's health infrastructure (technical platform failures, repeated strikes in university hospitals, incomplete free health care and the unavailability of medicines) and exacerbated by the security crisis that has been occurring the country since 2012. The lack of specialist doctors, combined with limited screening campaigns and a centralised and fragmented technical platform in Bamako, is particularly detrimental to breast cancer treatment. Women's lack of awareness, lack of information throughout the treatment process, stereotypes and opposition to amputations all play a significant role in their ability to seek and access quality care, leading some women to therapeutically wander and others to want to leave Mali. It also leaves them in debt and jeopardises the future of their children. However, the high level of trust in doctors, the involvement of international actors, the level of social support and the growing influence of civil society on the issue of cancer also represent great current opportunities to fight cancer in Mali.

**Conclusion:**

Despite the efforts of successive Malian governments and the commitment of international actors, the provision of health care is still limited in the country, entrenching global inequalities in women's bodies.

**Supplementary Information:**

The online version contains supplementary material available at 10.1186/s12885-024-11825-6.

## Introduction

Breast cancer is the most prevalent cancer in regard to incidence and mortality among women worldwide, including in Africa. In 2020, 2.26 million women were diagnosed with breast cancer worldwide [1], with 186,598 new cases and 85,787 deaths occurring in Africa [2]. The International Agency for Research on Cancer (IARC) predicts that the number of cancer cases will increase 50–60% over the next two decades. Indeed, there has been a very rapid increase in the incidence of breast cancer in Africa, particularly among women under 45 years of age [3]. The rapid increase in the number of new cases of breast cancer in sub-Saharan Africa (SSA) is partly explained by demographic changes (declining fertility and the ageing of the population) and lifestyle changes (decreasing breastfeeding, increasing obesity, physical inactivity, and alcohol consumption by women) [3]; further epidemiological research is underway. Improvements in cancer registration and the rapid development of diagnostic capabilities are also contributing to the increase of these figures.

In the field of cancer, SSA countries face structural challenges such as a lack of both infrastructure and material, technical, financial, and human resources. In certain West African countries, technical facilities do not offer services that can accomplish the complete therapeutic management of cancers, particularly given the absence of radiotherapy [4, 5]. At the same time, while some governments have made efforts to make chemotherapy widely available and free of charge (e.g., Côte d'Ivoire, Senegal and Mali), much remains to be done, including in terms of diagnosis and screening. There is limited evidence on the preparedness of health systems and women with breast cancer in West Africa.

Oncology is a relatively recent discipline in many West African countries, particularly in Mali. Mali is a large Sahelian country with a low-income economy and rapid population growth, with a fertility rate of 6.3 children per woman [6]. Mali has experienced instability and conflict since the 2012 military coup and the occupation of the northern regions by armed groups [7]. Forty-nine percent of Malians live below the extreme poverty line [8]. Women with breast cancer in Mali are particularly young (median age of 45 years [9], compared with 63 in France [10]), and the stage of the cancer is often locally advanced when they enter the formal care system (the biomedical area) [9, 11]. These late diagnoses can be explained by a number of factors, such as a low level of awareness of the disease among women and communities; rare screening policies; reliance on traditional medicine; the cost of medical care, transport and accommodation for women living outside the capital cities; the lack of specialist doctors, infrastructure and equipment; and mistrust of the medical establishment [9, 12–14]. A cancer registry was established in 1986 and covers the population of Bamako and the surrounding area. A total of 1,545 cancer cases were recorded in the district of Bamako in 2019, of which 1,017 were women (65.8%). Breast cancer was the most common type, with 294 cases (19.0%) [15].

A cancer plan was drawn up in 2007 but has never been implemented, mainly because of the political crises that have been ongoing since 2010. Cancer is now an integral part of noncommunicable disease (NCD) policy. The budget for cancer is part of the overall budget for NCDs and is mainly used to fund radiotherapy and chemotherapy services. [16]. The cost of the national strategic plan for the fight against NCDs for the 2019–2023 period covers various areas, such as registration, diagnosis, prevention, treatment, and the training of health care professionals [16]. Mali has been implementing public social protection policies in the health field for several years [17]; however, highly specific and expensive cancer care is not included in these measures. The various social protection policies are still very fragmented, and the policy that explicitly concerns those who are worst-off faces many funding and implementation challenges, thereby reducing the effectiveness of free health care [18]. Mali's health system remains underfunded, universal health coverage is very low, and catastrophic health spending is high [19].

However, little is known regarding the concrete state of cancer care infrastructure and oncological practices in this country. Thus, the aim of this article is to describe the challenges of access to oncology care in Mali to better understand the situation and inform health policy-makers and other stakeholders.

### Methodology

We used a qualitative approach that combined observations and in-depth interviews. We present our methodology following the Consolidated Criteria for Reporting Qualitative Research (COREQ) [20].

### Research team and reflexivity

#### Personal characteristics and relationship with participants

Two people were involved in data collection, namely, a Malian health anthropologist (AC), who is a university lecturer with considerable experience in interviewing people about sensitive issues such as illness, gender and sexuality, and a French sociologist (CS), who has been conducting field research and interviews with caregivers and women in Mali for 10 years. The sociologist also has a background as a midwife in France, with some experience in West Africa. The interview grid was shared, and the first interview was conducted with both individuals to ensure that we had a common understanding of the questions.

### Study design

#### Theoretical framework

**Fig. 1:**
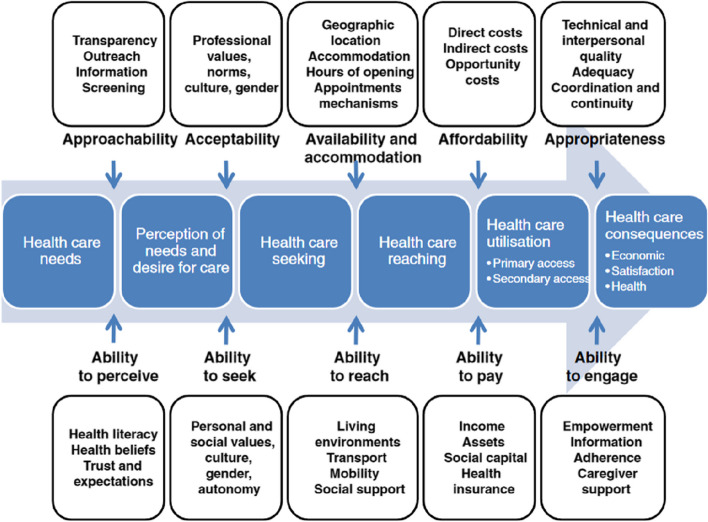
Levesque et al.’s definition of access to health care [21]

We used the theoretical framework developed by Levesque et al. [21] regarding access to health care (Fig. 1). We used this framework a posteriori to organise and analyse collected data [22]. Access is considered the possibility of identifying one’s health care needs, seeking health care services, reaching health care resources, obtaining or using health care services, and being offered services appropriate to one’s needs for care [21]. The results will therefore be presented according to the five dimensions of accessibility of services conceptualised by Levesque et al.: 1) approachability; 2) acceptability; 3) availability and accommodation; 4) affordability; 5) appropriateness, as well as according to the five corresponding abilities of persons interact with the dimensions of accessibility to generate access: 1) ability to perceive; 2) ability to seek; 3) ability to reach; 4) ability to pay; 5) and ability to engage.

#### Participant selection

The research took place in Bamako between July 2021 and July 2022. Health professionals, women, and associations were purposively recruited [23]. Thirty-eight semistructured interviews were conducted with health professionals treating cancer in Mali (*n* = 10), with women suffering from breast cancer (*n* = 25), and with representatives of associations (*n* = 3).

The recruited health professionals represented key players in cancer care in Mali: oncologists (*n* = 3), surgeons (*n* = 2), public health doctors (*n* = 2), radiotherapists (*n* = 1), pathologists (*n* = 1), and psychologists (*n* = 1). There were 9 men and 1 woman. Women were recruited through cancer associations (*n* = 20) and doctors (*n* = 5). Finally, the association representatives (*n* = 3) included those from Mali's two main breast cancer associations and a government-recognised traditional healer association.

#### Settings

All interviews with health professionals were conducted in health care facilities. Interviews with women were conducted at home (*n* = 20) and in health facilities (*n* = 5). Interviews with association representatives were conducted at the associations' head offices.

#### Data collection

The interviews were conducted using interview grids for health professionals (Additional file 1) and women (Additional file 2). The grids consisted of open-ended questions. The following topics were covered with the health professionals: the existence of professional training in oncology in Mali and their personal career paths (at the national and international levels); the cancer care offered in Mali (available human resources, infrastructure, key national and international actors, health policies, obstacles and challenges); and the therapeutic mobility of health professionals and women (incoming and outgoing). During the interviews with women, the first part retraced their care pathway, including the first signs, the various recourses (formal and informal), contacts, the information received, relations with health care professionals, difficulties, costs, and mobility. The second part was devoted to their relationship with their body and representations of 'femininity' (i.e., what it means to feel like a 'woman') in the context of the disease, including the effects of treatment on the body, the experience of amputation, self-image, sexuality, conjugality, perceived discrimination, the unveiling of the body, and relationships with other women (particularly possible cowives). Across these two major themes, the issue of biographical breakdowns was explored (marital, professional, and social breakdowns). Finally, the interviews with association representatives reviewed the history of the association's creation and then utilized combined questions from the interview grids for health professionals and women; the selected questions addressed the provision of care, care pathways, and women's life paths.

The median duration of the interviews was 61 min with the health professionals, 70 min with the women, and 38 min with the association representatives. The women’s ages ranged from 30 to 70 years (median age 43 years), with various backgrounds (housewives, doctors); they all lived in Bamako but came from different regions of Mali and different ethnic groups. Most interviews were recorded and transcribed (*n* = 34/38). Four interviews were not recorded because the interviewees refused (all were health care workers). Notes were taken during these interviews and the participant observations. This process produced a corpus of data alongside the transcripts of the interviews.

The French sociologist carried out participant observations (*n* = 40) in the oncology department of a university hospital in Bamako during medical consultations and chemotherapy treatments in July 2022. The researcher not only took part in these consultations, helping to measure blood pressure, oxygen saturation, and weight, but also took free-style notes in a notebook.

### Analysis and findings

#### Data analysis

Data analysis was conducted using a comprehensive approach to analyse experiences as they were lived and not necessarily as they objectively occurred [24]. The analyses were carried out manually by the two researchers who collected the data. These researchers carried out the content analysis of themes and subthemes independently before comparing their results, which were consistent. The results were then presented in October 2022 by the Malian anthropologist at three result-reporting sessions held in Bamako. Caregivers, women, patient associations, and political decision-makers were present at these sessions (60 people). The participants were enthusiastic and involved in the sessions. They asked many questions and added their own remarks and comments to the discussions. They all approved and improved the results of the research.

#### Reporting

To add transparency and trustworthiness to our findings and interpretations of the data, we will include quotations from different participants in the results section.

## Results

The observations and interviews highlighted various obstacles to access to oncology care in Mali (Table 1).

**Table 1:** Barriers and opportunities to access oncology care in Mali for women with breast cancer

**DEMAND**	**Ability to perceive**	**Ability to seek**	**Ability to reach**	**Ability to pay**	**Ability to engage**
Opportunities		Confidence in doctors			Social support and association of patients
Barriers	Lack of information about cancer Representations and beliefs about breast and cancer Confusion surrounding the disease during pregnancy and breastfeeding	Therapeutic wandering Plans and influences of family members to seek treatment abroad Refusal to be amputated	Wandering from one hospital to another Cost of travel and staying in Bamako Cost of back and forth between hospitals The importance of the social network in reaching the top of the health pyramid	Very high cost of examinations and treatment in relation to average salaries	Lack of information about breast reconstruction Redhibitory cost of reconstruction
**SUPPLY**	**Approachability**	**Acceptability**	**Availability and accommodation**	**Affordability**	**Appropriateness**
Opportunities			DU^a^ in oncology since 2021	State funding for chemotherapy drugs and radiotherapy MSF^b^ funding for palliative care and treatment for localised breast cancer	Postmastectomy surgical reconstruction available
Barriers	Limited access to health care facilities in insecure areas Limited screening campaigns Limited number of mammography machines in public hospitals	Lack of communication between carers and patients about diagnosis and treatment Discrimination in access to information based on social class	Few specialised doctors Repeated strikes in public hospitals Technical platform centralised in Bamako and spread over different establishments Dilapidated state of operating theatres and lack of available equipment Only one radiotherapy machine with many breakdowns Frequent chemotherapy drugs stock-outs Targeted therapy (trastuzumab) not available	Incomplete free health care	Misdiagnosis and inappropriate prescriptions received before reaching specialists Lack of breast implant for reconstruction

^a^*DU* Diplôme Universitaire

^b^*MSF* Médecins Sans Frontières

### Approachability and ability to perceive

#### Insecurity

The conflict and insecurity that have been ongoing in Mali since 2012 were heavily present in the discussions and constitute an important barrier to access to health care, especially for women living outside the capital of Bamako. Mali's health system was already fragile before the security crisis began but has worsened over the past decade. According to the World Health Organisation (WHO), 116 health centres have been closed across the country due to violence; those that remain open struggle to function due to a lack of resources and qualified staff being regularly targeted by armed groups [25]. The conflict is disrupting access to health care with massive population movements, and the most qualified health workers refuse to travel to these conflict zones [26], complicating access to oncology care. Moreover, insecurity in the country makes it not only challenging to carry out awareness and screening campaigns in several regions of Mali but also difficult to reach women outside of Bamako.

#### Limited screening campaigns

In 2017, Mali launched a free public early detection campaign for breast cancer, implemented by the NCD Division of the Ministry of Health through the "Weekend 70" project (supported by the Orange Foundation) [16]. However, these campaigns reach a limited number of women, and the limited number of mammography machines available in public hospitals hampers these screening campaigns [16].

#### Lack of awareness, representations, pregnancy, and breastfeeding

The first sign perceived by women is almost always described in the same way: they mention a lump ("kourou" or "kourouni" in Bambara) appearing in the breast or under the armpits and sometimes a heaviness in the breast. This is the most common trigger for seeking treatment. However, many women explained that before they were confronted with this disease, they had never heard of cancer. Those who did previously know about the disease had a sister or friend affected or heard about it most often on the radio. The breast is symbolically charged; as some pointed out, "The woman's soul is in her breast" (healthworker 1) and "A woman's life is in her breast" (healthworker 2). A common social conception about a woman with a breast abnormality is that she has been cursed. The word “cancer" in Bambara is "baw/bon", which means "spell". This lack of information and these beliefs hinder the ability to perceive the disease:

“In fact, my older sister had the same illness and didn't survive. I got it into my head that when you have that disease you're not going to survive. In our society, when people say you've got cancer, they call it M'bo (a curse cast by a fetishist), so my sister went off into the bush to try and find a cure for it. By the time she got there to look for treatment, it was too late. When she came back, she died shortly afterwards” (Fifi, 47 ans).

Many stories of pregnancy and breastfeeding delay treatment and diagnosis and are confusing:

"The illness started 6 years ago. The illness started with a little lump on my breast that I would show people to say that there was a lump on my breast. It turned out that my child was still suckling, so he was easily weaned because the breast no longer contained any milk. I didn't have any pain in my breast, but the lump was as if you'd put a stone in my breast. So people said it was a bad spell, and we started the traditional treatment" (Awa, 41).

Many of the women's stories intertwine these different obstacles, with years of therapeutic wandering before arriving at a cancer treatment centre.

### Acceptability and ability to seek

#### A lack of information throughout the care process

The interviews highlighted a lack of information among many women. This lack of information affects their understanding and management of the disease. Some mentioned that the word "cancer" had never been mentioned by their carers. Observations and interviews held with women have shown that women are often left out of the diagnosis. For example, the pathology reports’ envelopes are closed and opened by the doctor and not by the woman herself. The disease is often shrouded in secrecy and taboo:

"Until now, no one has told me that I have cancer... I'm treated where people with cancer are treated, so I know what it is" (Olie, 70).

There is sometimes discrimination in access to information based on social class:

“Yes, and I often ask when I don't understand, but Dr. X doesn't really like being asked, because he told me that I didn't go to medical school, so I shouldn't try to unravel all the medical terms” (Coumba, 30).

However, women who have been given information are better able to cope with the treatments and their side effects:

"I had been warned [about the effects of chemotherapy] well beforehand. So I didn't feel at all bothered by the treatment. Because I was warned" (Koro, 61).

#### Wandering around

As previously mentioned, even before the diagnosis is well established, there is a whole period of therapeutic wandering during which women seek explanations for the signs they perceive. They go from centre to centre, sometimes consulting traditional healers as a first resort:

"I went to 5 small health centres, and they told me I had nothing. At the fifth centre, there was a pharmacy and I explained to them that I had two little lumps on my breast and they reassured me that it wasn't serious. So when I got home, I self-medicated" (Dili, 43).

#### Leaving Mali

The idea of seeking treatment in another country is a factor that comes into play very soon after diagnosis. This idea is often put forward by family or friends, who directly advise women to leave. However, while almost all the women in our interviews had thought about leaving at one time or another (and some had even tried to do so), the majority had stayed in Mali. Such women often stay on the advice of doctors who point to the existence of a health care system that is being upgraded day by day:

“[I wanted to go to France] because at first people told me I could find good treatment there, but now I tell people that we have the best doctors in the world. There's a woman I know who started her treatment in Tunisia but is now continuing with us. She paid 50,000 francs every Thursday. She has even said that she threw her money away because we can do it here” (Tou, 49 ans).

#### Refusal to be amputated

The term 'amputation' was a recurring theme in the women's speeches. This is why we use it again in the article when talking about the women's experiences. All the women spoke of the shock they felt when they were told that their breast would have to be amputated. Many said they thought they would never be able to live with just one breast; some doctors said that some women refuse treatment, saying they would "rather die with their 2 breasts than live with just one". Several women (or their husbands) initially refused to have their breast amputated (a radical mastectomy):

“I didn't want to lose my breast. I thought that at [hospital 1] it wouldn't be the same. I didn't tell the doctor at [hospital 1] that I'd left [hospital 2], that I’d left all the tests at [hospital 2] to start again there. But they also told me that the breast had to be amputated. So I left [hospital 1] to turn to traditional treatments. The traditional therapist told me that there was a bone in my breast, and he gave me an appointment to remove the bone in my breast” (Sira, 43 ans).

#### Confidence in doctors

Confidence in Malian doctors was very present and directly linked to acceptance of treatment or radical mastectomy:

"I trust Malian doctors completely; they have my full confidence" (Koro, 61).

"Our doctors are very good because they can achieve good results with very little money. My parents told me to go abroad because they knew that our doctors don't have all the resources. But I stayed, and after my operation, the wound healed in less than 15 days. I even took a photo and sent it to my daughter, who couldn't believe her eyes. She came and touched my breast and told me she'd seen wounds heal in France, but my wound looked just as good. ‘Your operation is really clean’. They're really good. Our doctors are really good. With the limited resources they have, they are very efficient and often help out financially with illnesses" (Satourou, 56).

Most doctors treating cancer in Mali are men, but this did not pose any problems for the women we interviewed.

### Availability, accommodation and ability to reach

#### Few specialised doctors

Mali's first medical oncologist has been practising in Bamako since 2012. In 2022, there were five oncologists working in the capital, all of whom were trained abroad (Morocco (*n* = 3), Congo (*n* = 1), France (*n* = 1)). There is no university diploma (DU) in general oncology, but a DU in senology was created in 2021.

“We have five oncologists, so we're not much compared to the population. We may need thirty or so to see if we can even relocate to other regions, because the regions are so far apart that bringing a cancer patient from Gao to be treated in Bamako… it would really be ideal if there was a mini-team to take the first steps and then, if things weren't going well, they brought him here. But for the moment, we're working like this here. So everyone is in Bamako” (healthworker 2).

#### Repeated strikes

Unlimited strikes regularly paralyse the Point G and Gabriel Touré University hospitals [27] and were very present during our fieldwork. These strikes lead to a halt in doctors' clinical activity and delays in treatment. The main demands are improved technical facilities, better hospital conditions for patients, transparent management of hospital resources, the reinstatement of doctors' bonuses, proper promotion for hospital contract staff, and respect for trade union freedoms.

#### A centralised and fragmented technical platform in Bamako

Cancer treatment in Mali is centralised in the capital of Bamako. Four hospitals (Centre Hospitalo-Universitaire—CHU Point G, CHU Gabriel Touré, and Hôpital du Mali, which are public hospitals, and Hôpital Mère Enfant du Luxembourg, which is a private hospital) provide most of the care. This care is spread out over several different establishments. While surgery is provided at all four sites, chemotherapy is often provided at the CHU Point G and the Hôpital Mère Enfant du Luxembourg. Radiotherapy is only available at the Hôpital du Mali, which has the only machine in the country. As a result, most women must navigate between these hospitals, incurring significant expenses and sometimes wandering from one treatment to another.

“This trip is very expensive for them. To leave their original living environment within Bamako itself, the palliatives there, the people who are hospitalised, in most cases, they can't get on a motorbike like most Malians. They have to borrow a vehicle. And if they don't have one at home, they must go by taxi. The minimum to get up here is 3,000 francs. If you're behind the river, for someone to cross, whether you want to or not, it's 4,000 francs minimum. So there and back it's already 9,000-10,000 francs. But, if you know that it's even worse with smelly sores, you'll pay 10,000 francs” (healthworker 3).

This fragmentation of cancer care settings explains why the pathways are so long and tiresome. Carers also complained about this fragmentation, explaining that they lose sight of certain women. They explained that women are confronted with different carers and different discourses and that they sometimes fall outside the realm of conventional medicine. Having a national oncology centre is now one of the main demands of careers and of cancer associations in Mali:

"So treatment is expensive, but if we had at least one centre, you'd just have to go to the centre and do the whole treatment. But if you have to go from area to area, it's not easy, especially for a patient" (Dili, 43).

#### Incomplete free health care and unavailable drugs

Since 2009, Mali has had a decree in place that makes chemotherapy free of charge in the country [28]. However, the observations and interviews revealed the frequent occurrence of drug stock-outs, often leading women and their families to buy chemotherapy products from private pharmacies. In addition, the products needed to administer chemotherapy (gloves, solution, needle, syringe) and the products prescribed to limit the side effects have to be paid for.

"Because, in most cases, they run out of stock. If that happens, you're going to find that it's not all of it, it's not all the products that are missing for chemo. But you will often find that the essentials are missing, or one of the products is missing. And the products that are missing, you have to buy them, and it costs around 50-60,000 francs to buy them" (healthworker 3).

In addition, a targeted therapy (trastuzumab), which is listed as an essential medicine by the WHO [29], is unavailable in Mali. This targeted therapy has dramatically improved survival rates in the subgroup of HER2-positive breast cancer.

#### Technical platform failing

Cancer treatment is based on a combination of surgery, chemotherapy, and radiotherapy. Surgeons deplored the poor state of operating theatres and the lack of available equipment:

"Perhaps this should also be noted in the problems we have with surgery, the shortage of consumables in the operating theatres; sometimes there isn't even any soap to wash with! That's the tragedy. Operations are postponed because of this, or because of nonsterile materials because our steriliser breaks down... the autoclave is broken down at the moment, so we have to go and sterilise the fields in Kati, at [hospital 2]. (...) Several times, the programme is stopped because of this! Also the operating table. I don't have a suitable table for reconstruction because the patient has to be put in a half-seated position. The table in our room dates back to ‘79” (healthworker 6).

#### The central issue of radiotherapy

Mali has 4 radiotherapists but only one radiotherapy machine for the whole country. Various countries have been involved directly (through purchases or donations) or indirectly (through training) in setting up and running radiotherapy to enable patients to benefit from this treatment; these countries are Austria, China, Morocco, Egypt, and Iran. Radiotherapy fees are subsidised by the State for Malian citizens, with only consultations subject to a charge (1,500 FCFA). Gynaecological cancers are the most frequently treated by radiotherapy. Since it became available in 2014, the machine has suffered several breakdowns, which have been regularly reported in the national media. Patients and carers deplore these repeated breakdowns, which interrupt treatment and contribute to long delays in obtaining appointments; patients' associations are mobilising to denounce the enormous delays that can occur before accessing this treatment [30]. The recurrent unavailability of this essential breast cancer treatment and the late stages at which women present explain why breast amputation (radical mastectomy) is almost systematic in Mali:

"Yes, normally this should be my eleventh session, but last Tuesday, the table was blocked. There was only one mechanic, but they called him in vain; he didn't pick up the phone. How can he play with people's lives? But why don't they train other people? I really don't understand. And even the machine has to be backed up; another one has to be put in at [hospital 1] or in [hospital 3]. Because we only have one machine for the whole of Mali. So if it's blocked, that's the end of it; it's used too much and that's why it breaks down so often. So we should have two or three; if it breaks down, it's the Egyptians who come to help. It's really not normal" (Mah, 51).

#### The importance of the social network in reaching the top of the care pyramid

The women's journeys showed that referrals to specialists higher up the health pyramid are often made through networks of acquaintances, underlining the social determinism involved in finding a resource person for this disease and accessing appropriate treatment:

"My younger brother works in a CSCom; he's a surgeon. I showed him my breast, and he told me he couldn't confirm that it was cancer, but to go and see someone. He even wrote a note for me to go to the hospital and see one of his colleagues" (Ili, 43).

### Affordability and ability to pay

#### Costs not covered by the state

Cancer treatment, which is very specific and expensive, is not included in the state's social protection measures, which many of the people we met complained about:

"It's not a disease for the poor, because the drugs are expensive. Although I'm covered by the AMO (Assurance Maladie Obligatoire), I had a scan that cost 60,000 francs because it was not covered by the AMO. That's not the only thing; I did it every quarter. Each quarter I paid 90,000 francs for scans, not counting the blood tests. The blood tests cost me 30,000 francs at PA&KA. I'm a civil servant, and everyone knows that a civil servant's salary can't cover this illness. But if I'm here today, I must first of all thank God and my doctors and also my parents who looked after me. If you don't have anyone to give you moral support, the disease will get the better of you, and I'm sure of that" (Lakaré, 46).

#### A disease that leads to debt and compromises children's futures

The question of the staggering cost of cancer was present in all the speeches. For women, it is a dramatic expense that leads to the sale of property (plots of land, shops) and the indebtedness of an entire family when it does not make treatment impossible. For carers, it is a major obstacle to caring for women and a source of suffering for them, too. They express enormous frustration at not being able to treat some women due to lack of resources. This frustration is one of the alleged reasons for leaving the country and working abroad. This was a key issue for all the actors involved; thus, it must be an absolute priority if we are to make progress on the cancer problem in Mali. The following point is one of the priority claims formulated by patients' associations in Mali:

"Yes, and it even affected my child's education, because I used to pay for home tuition, but I stopped because of my illness. Every time I went into debt, and I couldn't even manage to pay his school fees. I had a plot of land that I sold, and I used the money to pay for my treatment. I didn't ask anyone for a single franc. Not even my adoptive mother. I took taxis, and I had chemotherapy for 25,000 francs every 15 days, and that's not covered by the AMO. For me it was 25,000 francs, but for others it was 50,000 francs, not counting blood tests; so for others it was more than 100,000 francs every 15 days. But after the operation, the chemotherapy I had cost 35,000 francs at the Koulouba pharmacy. At the Mohamed V clinic or the PEWO, it cost 45,000 francs, and this was not covered by the AMO. The lack of money for treatment is why cancer kills so many people. I had my treatment with a man who suddenly disappeared. I asked him why he didn't come to the chemo anymore, because we'd had two rounds of chemo and we didn't see him again. He told me that he had sold all his herds, that he hadn't had the operation yet and that he had nothing left; so that's it, he can't come for chemo anymore" (Tou, 49).

#### Involvement of international actors

Some influential actors in the fight against cancer in Mali have positioned themselves to remove financial barriers, raising questions of sustainability and substitution. Médecins sans Frontières (MSF) has been active in Mali since 2018, with a significant program targeting palliative care and female cancers in particular [31]:

"Thank God I was taken care of by Médecins Sans Frontières; I really didn't spend any money on treatment" (Rokia, 60).

However, this program only treats the localised stage of breast cancer and not the metastatic stage, leading to misunderstandings among families and associations and frustration among carers:

"The other day, I was telling you that we had to do screening in Kati, and MSF told me that when there are cases there, treatment is free. If MSF manages to do that, it will really be a good thing. But the problem is, they say that... I've heard it said that they're going to take 100 women, if those women aren't going to die of the disease. If they know that those women aren't going to die… that's what I don't understand, that's what I don't understand. Only God knows about death! I lost my sister, my little milk sister, not four months ago, maybe three months ago, to breast cancer" (Fatou, 60).

### Appropriateness and ability to engage

#### Misdiagnoses

The interviews revealed numerous misdiagnoses and a tendency to minimalise symptoms during initial consultations:

"I asked my gynaecologist to do a mammogram and was told that the mammogram was normal apart from bilateral dilation, so I thought if it's normal why say ‘apart from dilation’? I was a bit puzzled by this term, so I said to myself that I had to go back. I told my gynaecologist that I wanted to go back, and the second time I was told that they thought it was a tumour, that it was benign. But the very fact of seeing a tumour made me feel a bit unsettled and I wanted to get to the bottom of it, so I asked for another mammogram and breast ultrasound, and this time it was confirmed". (Okia, 60).

Several women mentioned inappropriate prescriptions (ointments, various tablets, etc.), which often lead to treatment delays before reaching specialist doctors. For example, Ou, aged 44, 'went round and round' for three years before her cancer was diagnosed:

"I have a friend and her mum is a doctor. So one day, I showed her my breast, and she gave me an ointment and said if it didn't go away, to go to hospital. I called her and explained what was going on and she told me to go to hospital, so I went to the Csref. The doctor there gave me an ointment and some tablets, but with all that, the lump wouldn't go away. He prescribed for me a mammogram, and I went to the laboratory to have it done; I gave him the results, and he told me that the ointment would neutralise it. He's the one who misled me because he always insisted that it would neutralise... He never told me about the cancer. He told me it was a lump that he would neutralise with the ointments and tablets he gave me”.

#### Therapeutic mobility and cancer

The issue of cancer treatment in Mali is indicative of the current global health context [32], which is accompanied by multiple therapeutic mobility of people, products and knowledge [33]. Faced with an incomplete range of health care services or due to a lack of confidence in the Malian health care system, some women travel to access treatment, and some travel abroad. Carers pointed out that many women travel to Tunisia because, unlike Morocco, this country does not require a visa; thus, "it has become the Mali of overseas" (health professional 2). The interviews showed that the pursuit of cancer treatment in another town, another country, or even another continent is an important reality.

The current research on Mali has highlighted 4 levels of mobility that seem relevant to take into account. First is mobility at the national level, since all treatment is concentrated in the capital of Bamako, women often come from other regions for treatment. Second is mobility within the capital itself; since the various treatments are relocated within the city, women have to go from one hospital to another, which wastes time, energy and money, as we mentioned earlier. Then, there is South‒South regional mobility, with some women going to Senegal for treatment, for example. Finally, there is international South‒North mobility. Finally, it should be noted that Mali is not just an "outward mobility" country for cancer treatment; the country and its health care system are also a source of therapeutic attraction for neighbouring countries. This is the case, for example, for some women from Guinea Conakry, where oncology care is virtually nonexistent, and for women from Côte d'Ivoire, who are attracted by the lower prices in Mali and chemotherapy that is theoretically free.

#### The issue of surgical breast reconstruction

As part of a project launched in 2013, a French team (ICM-Institut Cancer Montpellier) regularly welcomes Malian surgeons from Point G to train them for a year in surgical breast reconstruction. As a result, a team of 5 surgeons is now offering postmastectomy surgical reconstruction in Mali. However, the team is faced with technical difficulties, such as the lack of breast implants:

"You can't find prostheses anywhere in Mali. Normally it's the patient who has to pay, but unfortunately, people don't know about it, they don't know... there are patients who are waiting, who are really ready to buy the prosthesis, but they can't find it here. It's also a product that requires a lot of traceability, everything has to be traced. So we need to find a distributor here. One of the laboratories—Johnson & Jonhson—is here and says it's available if we need it, so we can contact them.... but we're not doing much campaigning ourselves. I even wanted to do something, maybe list all the patients who have had a mastectomy, try to convince them to carry out a small interviewee survey, see roughly how many want a reconstruction, and then, depending on the results, maybe make a request" (healthworker 6).

The cost of reconstruction is the same as that for any other surgery (40,000 FCFA, including anaesthetic) at CHU Point G. Many women said they would like to have their breasts reconstructed, but most did not know that this type of surgery existed, while others said the cost was too high for them to be able to do it.

#### Social support and the growing influence of civil society on the issue of cancer

The role of peers in the reconstruction of the self after cancer is fundamental [34]; many women have testified to this. Powerful bonds are sometimes forged between patients. Various spaces were regularly mentioned as places where women in hospitals can get together, i.e., waiting areas and shared chemotherapy rooms. Other groups, such as social networks and associations, were also mentioned. Mocking, teasing, making fun of one's suffering and showing off one's body and scars were particularly explicit in the women's speeches. This social support contributes to real empowerment in enduring the disease and rebuilding oneself:

"I met a lady I didn't know who was having her first session. She came and sat down next to me and asked me, ‘Are you going to have chemo, too?’ I said, ‘No, it doesn't hurt’. She said, ‘I've heard that when you have chemo, it either kills people or makes the disease worse. In any case, chemo or no chemo, you die’. She cried, and I said to her, ‘Crying is not the answer; you do your treatment, and you'll see improvements’. She's improved a lot now, and from time to time she calls me up and says, ‘You gave me confidence for the chemo and the treatment; it's thanks to you that I'm here’. There are lots of women like that" (Fifi, 47).

Moreover, associations that fight cancer have recently begun to organise and structure themselves. For example, the ALMAC association was created in 2000 [35], and the Les Combattantes du Cancer association was created in 2016. Most of their work involves informing patients and their families, supporting patients and mobilising the community for screening. They work closely with health care professionals and humanitarian organisations involved in cancer care. They take centre stage in the media during major events such as Octobre Rose or World Cancer Day to raise awareness and inform the general public.

## Discussion

This research has shown that access to cancer care in Mali is difficult because of the fragility of the services offered and the low capacity of women and their families to engage in care, although opportunities are also present. Several sociohistorical factors, both locally and globally, contribute to this situation.

### Breast cancer: a disease underrepresented on the global political agenda

The fight against chronic diseases remains relatively marginal on the global health agenda despite the increase in the number of cases since the 1970s [36] and multiple calls to put noncommunicable diseases on the global political agenda [37, 38]. In terms of cancer research carried out for women in countries in the global south, cervical cancer accounts for the majority of research [37]. Breast cancer is often overlooked in the context of cancer research in Africa, even though this form of cancer accounted for 29.5% of all cancers affecting women on the continent in 2020, compared to 18.5% for cervical cancer [2]. This lack of attention is partly due to the link between cervical cancer and sexually transmitted diseases (specifically HIV and HPV) and funding opportunities in this area.

### The verticalisation of infectious diseases and the issue of universal health coverage

Research has shown that the provision of oncology services in Mali is fragile. It has also shown that the issue of cost is a major concern for women and their families, as well as for caregivers, because it creates a deep sense of unease. At both international and national levels, health care resources are still mainly focused on infectious diseases and maternal health, leaving aside the new priorities associated with NCDs [19]. Similarly, prevention remains underfunded in comparison with more curative and hospital-based approaches. The latest national health accounts for Mali relate to 2015 and were published in 2018 [39]. They show that preventive care providers account for only 9% of the total expenditure, compared with 35% for hospitals. In addition, infectious and tropical diseases account for 49% of the total expenditure, while NCDs account for only 15%. In the latter category, tumours account for 0.52% of the total expenditure, while noncommunicable diseases account for 3.4% (38% in Bamako).

### The challenge of radiotherapy

The issue of radiotherapy is a source of tension in several countries in the subregion, but the maintenance of this equipment and the demanding international standards associated with it make radiotherapy difficult to operate at the local level [5, 30, 40]. In Mali, following years of lobbying by cancer associations, the state financed a second state-funded linear accelerator in June 2022. Unfortunately, it was decided to remove the old machine to install the new one, as only one bunker was available at that time. Health professionals regret this decision and would have preferred to have another bunker, as having two machines would have enabled more patients to be treated. In addition, the installation of this new machine has faced many technical difficulties; one year after its purchase (June 2023), this machine was still not operational, leaving the Malian population without radiotherapy for more than 12 consecutive months.

### Social and association networks

Several respondents mentioned social networking and the internet as a way to raise awareness, including among men. Cancer is a sensitive issue, so the use of social networks is a popular and effective way to raise awareness. Mobile technologies have emerged as new tools for delivering health care and health-related services in the field of cancer, especially breast cancer. They relate to information on the disease and treatment, disease management, awareness raising and prevention [41]. In Mali, cancer information is disseminated mainly through videos, messages and pictures on Facebook and WhatsApp. Most of this information comes from unofficial or nonscientific sources, and the use of social networks to provide health information and services by public authorities and health professionals remains limited. Patient associations also use these communication channels to share their experiences and knowledge about the disease.

These associations also participated in the creation of the Malian League against Cancer (LIMAC) in 2021, bringing this public health issue to a political level. Les Combattantes is fighting for cancer to be registered with the AMO (Assurance Maladie Obligatoire) and for the creation of a national oncology centre to centralise care. There is now a real opportunity to support these local initiatives and to support civil society, health professionals and policy-makers by strengthening the link between science and society. Improving access to screening and treatment for cancer patients in Mali requires the development of knowledge transfer systems such as participatory research projects and partnership models for capacity building or planning and the implementation of cancer control strategies. Other African experiences have shown that collaborative partnerships involving frontline workers, patients, researchers and those involved in funding and supporting public health initiatives can enable local decision-makers to make informed, practical and culturally sensitive strategic decisions together on how best to implement cancer control strategies [42–44].

### A syncretism of medical resources

Almost all of the women consulted traditional healers during their treatment. The use of traditional healers and that of conventional doctors are not in conflict but rather complement each other, as already mentioned in the case of cancer treatment in SSA [45–47] and in Mali [13]. However, a loss of time was often mentioned in the interviews when using traditional healers, as documented in Bamako [12]. Traditional practitioners need to be trained to be aware of the urgent need to refer women with breast problems to a specialist centre. However, they should not be sidelined but remain involved in the holistic care of women who come to them [48].

### High level of trust in doctors

The women were almost unanimous in expressing a high level of confidence in their doctor, who has a strong influence on the decisions they make during their treatment. At first glance, this would seem to be in contrast to the much more well-documented and long-standing area of maternal health, where it is known that women are often subjected to mistreatment and even violence during childbirth [49–51]. The field of oncology is still relatively unexplored in West Africa; thus, the relationship between health professionals and patients could be further explored in the future to understand whether these relationships truly differ between these two health specialties and if so, why.

### Claim for a national cancer hospital

The fragmentation of cancer services in the capital was seen as a source of suffering for both carers and women. Carers lose sight of women as they wander geographically and find it difficult to get together for the multidisciplinary counselling sessions that are necessary to ensure the quality of women's care. Women waste time, energy and money travelling between hospitals. Several West African countries are opening or intend to open national oncology centres to avoid this wandering; Côte d'Ivoire opened the CNRAO (Centre National de Radiothérapie et d'Oncologie Alassane Ouatarra) in 2018 [47], and Benin is building an international reference hospital in Abomey Calavi [52]. However, related challenges still remain.

### Limitations and strengths

The use of a qualitative design gives us a rich and detailed picture of the topic. However, the restricted number of participants and settings covered makes it difficult to generalise the findings. An essential aspect of this research consists of the results reported in Bamako to women, health care professionals and political decision-makers. Our shared vision of the challenges to be met in terms of access to oncology care reinforces these results.

Using the conceptual framework of Levesque et al. [21] was very useful for analysing and ordering our results. Specifically, its a posteriori use was helpful in explaining the imbalance in the number of results between supply and demand in access to care (the results concerning demand being more represented).

## Conclusion

Breast cancer affects many women in Mali, and its incidence rate continues to rise. Despite the efforts of successive Malian governments and the commitment of international actors, the provision of health care is still limited in the country, entrenching global inequalities in women's bodies. Documenting breast cancer cases, the management of which is also impacted by the ongoing conflict in the country, makes it possible to highlight the difficulty in developing quality medical care in this area.

The inequalities and failures of health care systems in countries with limited resources largely explain the need for patients to travel to receive care [53]. This situation highlights the role and responsibility of nation-states as drivers of transnational therapeutic mobility [54].

### Supplementary Information

**Additional file 1: Appendix 1. **Interview Guide Caregivers English.

**Additional file 2: Appendix 2. **Interview Guide Women English.

## Data Availability

The datasets generated and analysed in the current study are not publicly available due to their sensitive nature and the fact that the interviews are pseudonymised but not fully anonymised. However, they are available from the corresponding author upon reasonable request.
