# Supplementary material for: Access to oncology care in Mali: a qualitative study on breast cancer
Source: BMC Cancer. 2024 Jan 15;24:81. doi: 10.1186/s12885-024-11825-6 (PMC10788985; doi:10.1186/s12885-024-11825-6)
Supplement: Supplementary file 1 — Additional file 1: Appendix 1. Interview Guide Caregivers English. [file 12885_2024_11825_MOESM1_ESM.docx]

**Caregiver interview guide**

1) Information on confidentiality, anonymity and registration

Reading or handing over of the information sheet and the consent form. Request for agreement before registration.

- Can you give me a first name you like? This will be your pseudonym during our survey.

2) Main information about the interviewee

- Before starting the interview, can you introduce yourself in a general way?

Relaunch:

- Where and in which year have you been born?

3) Professional training in oncology and personal background (national and international)

- Can you tell me how long have you been practicing oncology? Where did you study?

Relaunch:

- Did you go to a foreign country during your training?

- What are the possible training courses in oncology in Mali?

4) Supply of cancer care in Mali (available human resources, infrastructure, key national and international players, health policies, barriers and challenges)

- Can you tell me about breast cancer in Mali? What is it like to have breast cancer here?

- What are the treatment options in the country?

- Are there any NGOs that cover some of the costs or provide support?

Relaunch:

- Are there many oncologists? Radiotherapists? Surgeons? in the country

- Which hospitals treat breast cancer in Mali?

- Is it possible to have breast reconstruction with surgery?

- What is the cost of treatment?

5) Women's therapeutic mobility (inbound and outbound)

- Do some women go to another country for treatment?

- Do some women come from another country for treatment in Mali?
