# Supplementary material for: Access to oncology care in Mali: a qualitative study on breast cancer
Source: BMC Cancer. 2024 Jan 15;24:81. doi: 10.1186/s12885-024-11825-6 (PMC10788985; doi:10.1186/s12885-024-11825-6)
Supplement: Supplementary file 2 — Additional file 2: Appendix 2. Interview Guide Women English. [file 12885_2024_11825_MOESM2_ESM.docx]

**Interview guide for women**

1) Information on confidentiality, anonymity and registration

Reading or handing over of the information sheet and the consent note; request for registration

Can you give me a first name that you like? This will be your pseudonym during the survey.

2) General information about the respondent

- Before starting the interview, can you introduce yourself in a general way?

Relaunch:

- Where and when were you born? Where did you grow up?

- Are you married? Do you have children? Do you work?

3) Care pathway: the first signs, the various care recourses - formal and informal -, resource persons, difficulties, information received, relations with health professionals, mobility

- Can you tell me about your illness?

Relaunch:

- When did you feel the first sign that worried you?

- What did you think about?

- Who did you talk to at first?

- Where and who did you consult? What were you told? Please tell me about the different places you went, the different people you saw for your care ?

- Who helped you around you in these different stages?

4) Relationship to the body and representations of "femininity" (i.e. what it is to feel "woman") in the context of the disease: the effects of the treatments on the body, the experience of breast amputation, self-image, sexuality, conjugality, perceived discrimination, unveiling of the body, relationship to other women

- Can you tell me about your relationship with your husband?

Relaunch: : did your husband support you?

- What was your husband's reaction?

- How do you feel about your body? Can you look at yourself in the mirror?

- Can you tell me about the effects of the treatments on your body?
